# Rapid and Highly Sensitive Detection of Variant Creutzfeldt - Jakob Disease Abnormal Prion Protein on Steel Surfaces by Protein Misfolding Cyclic Amplification: Application to Prion Decontamination Studies

**DOI:** 10.1371/journal.pone.0146833

**Published:** 2016-01-22

**Authors:** Maxime Belondrade, Simon Nicot, Vincent Béringue, Joliette Coste, Sylvain Lehmann, Daisy Bougard

**Affiliations:** 1 Laboratoire TransDiag, UMR 1058, Etablissement Français du Sang Pyrénées-Méditerranée, Montpellier, France; 2 Institut National de la Recherche Agronomique, UR892, Virologie Immunologie Moléculaires, Jouy-en-Josas, France; 3 CHRU de Montpellier and Université de Montpellier, IRMB, INSERM U1183, Laboratoire de Biochimie Protéomique Clinique, Montpellier, France; University of Maryland School of Medicine, UNITED STATES

## Abstract

The prevalence of variant Creutzfeldt-Jakob disease (vCJD) in the population remains uncertain, although it has been estimated that 1 in 2000 people in the United Kingdom are positive for abnormal prion protein (PrP^TSE^) by a recent survey of archived appendix tissues. The prominent lymphotropism of vCJD prions raises the possibility that some surgical procedures may be at risk of iatrogenic vCJD transmission in healthcare facilities. It is therefore vital that decontamination procedures applied to medical devices before their reprocessing are thoroughly validated. A current limitation is the lack of a rapid model permissive to human prions. Here, we developed a prion detection assay based on protein misfolding cyclic amplification (PMCA) technology combined with stainless-steel wire surfaces as carriers of prions (Surf-PMCA). This assay allowed the specific detection of minute quantities (10^−8^ brain dilution) of either human vCJD or ovine scrapie PrP^TSE^ adsorbed onto a single steel wire, within a two week timeframe. Using Surf-PMCA we evaluated the performance of several reference and commercially available prion-specific decontamination procedures. Surprisingly, we found the efficiency of several marketed reagents to remove human vCJD PrP^TSE^ was lower than expected. Overall, our results demonstrate that Surf-PMCA can be used as a rapid and ultrasensitive assay for the detection of human vCJD PrP^TSE^ adsorbed onto a metallic surface, therefore facilitating the development and validation of decontamination procedures against human prions.

## Introduction

Transmissible spongiform encephalopathies (TSEs), or prion diseases, are fatal neurodegenerative disorders of both humans and animals. The key event in TSEs is the post-translational modification of the host-encoded cellular prion protein (PrP^C^) into an abnormal aggregated isoform (PrP^TSE^), which accumulates in the tissues of infected individuals. PrP^TSE^ itself is believed to be the infectious agent, or the most reliable disease marker, according to the widely accepted “protein-only” hypothesis [1–3]. Prions exhibit strain diversity in the same host species similar to conventional pathogens, with each strain characterized by specific and heritable phenotypic traits. Compelling evidence indicates that these strain-specific properties are encoded within structural differences in the conformation/aggregation state of PrP^TSE^ [4–8].

TSEs have been widely investigated since the emergence of the bovine spongiform encephalopathy (BSE) epidemic in cattle, with convincing scientific data on its transmission to humans in the form of variant Creutzfeldt-Jakob disease (vCJD) [9, 10]. A total of 229 vCJD cases have been reported worldwide to date, with the vast majority in the UK and France (177 and 27 cases, respectively) [11]. The incidence of vCJD strongly declined after peaks in 1999 and 2004 in the UK and France, respectively. However, concerns have been raised that secondary iatrogenic propagation of vCJD in humans could occur due to the presence of PrP^TSE^ discovered in nervous tissues as well as lymphatic tissues and blood [12–14]. In addition, the prevalence of asymptomatic individuals infected with vCJD remains extremely uncertain, although it has been estimated that 1 in 2000 people born between 1941 and 1985 in the UK are potentially infected by prions, according to recent surveys of archived appendix tissues [15].

The unusual resistance of prions to either chemical or thermal inactivation [16, 17] poses a serious threat to the control of infection in healthcare facilities. Moreover, prions readily bind to many surfaces, especially stainless steel, rendering decontamination of prion-contaminated surfaces particularly difficult. For this reason, decontamination reference procedures include the use of high concentrations of either sodium hydroxide or sodium hypochlorite. However, the incompatibility of these harsh chemicals with various medical devices has necessitated the development of specific prion decontamination processes to ensure the reprocessing of fragile instruments. The efficiency of these processes to decontaminate and inactivate prions requires thorough validation, which remains an important issue. Currently, validation relies on animal-intensive, time-consuming and expensive bioassay infectivity studies using non-human prion strains, i.e. hamster- or mouse-adapted scrapie prions. However, studies have shown that inactivation procedures validated using rodent prions cannot be fully extrapolated to human prions [18, 19]. As an example, human sporadic CJD (sCJD) prions were shown to be 100,000 times more resistant to acidic sodium dodecyl sulfate inactivation than hamster Sc237 prions [18].

The aim of this study was to develop a rapid, cost-effective and ultrasensitive method for the detection of abnormal human prions adsorbed onto a surface while avoiding the extensive use of laboratory animals. We focused on protein misfolding cyclic amplification (PMCA), a prion amplification technology [20, 21]. PMCA reliably replicates mammalian prions *in vitro*, allowing amplification of minute amounts of PrP^TSE^ to levels detectable by conventional biochemical techniques. We used “Surf-PMCA”, an assay based on seeding PMCA reactions with prion-contaminated surfaces (steel wires). The capacity of various prion decontamination procedures, including reference treatments and prion-specific marketed reagents, to remove sheep scrapie prions (127S strain) and human prions (vCJD) from stainless steel surfaces were assessed *in vitro* with high sensitivity. The Surf-PMCA method presents many potential advantages in comparison with existing decontamination validation methods, including a broader detection range and the possibility to test the decontamination of genuine human prions.

## Materials and Methods

### Infectious material

A 10% (w/v) 127S-scrapie infectious brain homogenate (127S-IBH) (INRA, Jouy-en-Josas, France) was prepared from transgenic mouse brains (tg338 line) [22] infected with the 127S prion strain and collected in the terminal stage of the disease [23]. All the animal experiments made to inoculate the mice and collect the brains at euthanasia were carried out in strict accordance with EU directive 2010/63 and were approved by the INRA institution local ethics committee (Comethea; permit number 12/034).

vCJD infectious brain homogenate (vCJD-IBH) and human normal brain homogenate (hu-NBH) were obtained from the UK National Institute for Biologicals and Standards (NIBSC) CJD Resource Centre (www.nibsc.ac.uk/cjd/brainsamples.html) in the form of 10% (w/v) homogenates in 0.25M sucrose (NIBSC ref materials: vCJD, MM, (NHBYO/0003, designated Blue) and CJD control (NBHZO/0005, designated Clear). No IRB or ethics committee was required. Human tissues were anonymized.

### Preparation and contamination of stainless steel wires

Stainless steel wires (FE 245102 –Goodfellow, England; diameter 0.15 mm) were cut into small 3 mm pieces. They were washed in pure ethyl acetate for 15 min under constant sonication (BPAC, France), washed in 2-propanol, rinsed in normalized water (NF-EN14476), washed in 2% Triton-X100 for 15 min under constant sonication, rinsed in normalized water and dried for 24 hr at room temperature (RT).

For prion contamination before treatments, wires were incubated in 96-well plates (one wire per well) containing 80 μl of 1% 127S-scrapie or 10% vCJD brain homogenates for 5 min under constant shaking at 300 rpm at RT in a thermomixer (Eppendorf). The same 80 μl of IBH was re-used serially to contaminate up to 8 wires. To evaluate Surf-PMCA sensitivity, wires were contaminated with 80 μl of serial dilutions of 127S in 10% tg338 normal brain homogenate (10^−2^ to 10^−9^) or vCJD-IBH in 10% humanized transgenic mice (tg650 line) [24] brain homogenate (tg650-NBH) (10^−1^ to 10^−9^). For negative controls, wires were incubated in 80 μl of 10% tg338 NBH or 10% hu-NBH. After air-drying overnight at RT, wires were rinsed with PBS for 5 min under constant shaking; air-dried and 96-well plates were sealed for storage at -80°C prior to processing and PMCA amplification.

### Processing of contaminated steel wires

Before processing, 96-well plates containing contaminated wires were dried at RT. Wires were then exposed to the different formulations or procedures for prion disinfection listed in Table 1. They included: control treatments of water (H_2_O) and 1.2% (v/v) peracetic acid (PAA) for 60 min; "standard" decontamination procedures; marketed reagents previously validated by the French Regulation Agency (ANSM) (S1 Table) anonymized *A* to *F*; and finally treatment with 0.2% SDS / 0.3% NaOH for comparison with published data [25]. Except for some marketed reagents, all reference chemical treatments were performed at room temperature.

**Table 1:** Prion decontamination procedures tested in this study.

**Inefficient treatments**	Water (H_2_O)– 60 min
	Per acetic acid (PAA) 1.2%– 60 min
**“Standard” partially efficient treatments**	Steam sterilization 121°C– 20 min
	Sodium hydroxide (NaOH) 0.1N—15 min
	Sodium hypochlorite (NaClO) 0.2%– 15 min
**“Standard” fully efficient treatments**	Steam sterilization 134°C– 20 min
	NaOH 1N—60 min
	NaClO 2%– 60 min
**Treatment under development**	Sodium dodecyl sulfate 0.2% / NaOH 0.3%– 10 min
**Anonymized marketed treatments**^a^	*A*
	*B* (1X)*–B’* (2X)
	*C*
	*D*
	*E*
	*F* (2X)*–F’* (1X)

^a^marketed treatment have been validated by the French regulation Agency (ANSM) (S1 Table)

Test wires in 96-well plates (one wire per well) were incubated in 200 μl of the different disinfectant solutions and treated according to the manufacturer's instructions. Two different conditions were tested for two of the reagents (Table 1*B* and 1*F*). For steam sterilization, wires were placed in a ceramic plate. After treatments (except steam), all wires were rinsed under constant shaking three times with 200 μl of normalized water for two min at RT and air-dried in 96-well plates for at least 2 hr. Finally, 96-well plates were sealed and stored at -80°C prior to PMCA amplification.

### Protein misfolding cyclic amplification (PMCA)

Normal brain homogenates (NBH) from either human PrP (M^129^ allele, tg650 line) [24] or ovine PrP (VRQ allele, tg338 line) [22] transgenic mice that over express PrP^C^, 6 and 8 times respectively, were used as substrates for PMCA. After collection, mouse brains were rinsed in cold PBS and immediately frozen on dry ice before long-term storage at -80°C. Brains were then homogenized at a 10% (w/v) concentration in a conversion buffer composed of 150 mM NaCl, 1% Triton X-100 and protease inhibitor cocktail (Roche) in PBS (pH 7.2). Homogenates were clarified at 2000 x *g* for 20 seconds and frozen at -80°C in single-experiment aliquots.

Wires were inserted into 0.2 ml PCR-tubes and mixed with 90 μl of 7.5% NBH in conversion buffer for Surf-PMCA amplification (Misonix 4000, N.Y., USA). One PMCA round was composed of 80 cycles of 20 seconds of sonication at 220–240 W power followed by 29 min 40 seconds of incubation at 37°C. Ten μl of amplified product was mixed with 90 μl of fresh NBH in conversion buffer and subjected to an additional PMCA round of 80 cycles. Three or four rounds were performed depending on the prion strain.

### Proteinase K (PK) digestion and SDS-PAGE/Immunoblotting

Methods were performed as described previously [26]. Briefly, 20 μl of each amplified product was incubated at 45°C with PK (200 μg/ml) for 1 hr before denaturation at 100°C in SDS-PAGE sample buffer. Samples were run on 12% NUPAGE gels and electrotransferred onto PVDF membrane. Western blot (using the SNAP ID system, Millipore) was performed using 3F4 or 6D11 anti-PrP monoclonal antibodies for human and sheep prion detection, respectively (Eurogentec, 49001 Angers, France), and anti-mouse IgG peroxidase-linked secondary antibody (GE Healthcare, UK) linked to a chemiluminescent reaction (ECL blotting detection reagent, GE Healthcare life sciences, Amersham, France).

## Results

In this study we developed an innovative method for detecting prions on steel surfaces (Surf-PMCA), based on protein misfolding cyclic amplification (PMCA) technology. Steel wires are considered as relevant carrier models for surface prion disinfection studies by mimicking the contamination of surgical instruments by prions [27–29]. Initial optimization of the Surf-PMCA method was conducted using brain material (normal and 127S scrapie-infected brain homogenates) from transgenic mice (tg338) overexpressing ovine PrP^C^ (VRQ allele) [22, 23]. The method was next applied to human vCJD prions by using a reference brain homogenate from a patient with vCJD and normal brain homogenates from transgenic mice (tg650) overexpressing human PrP^C^ (Met129) as substrates [24].

### Optimization of wire contamination

**Fig 1:**
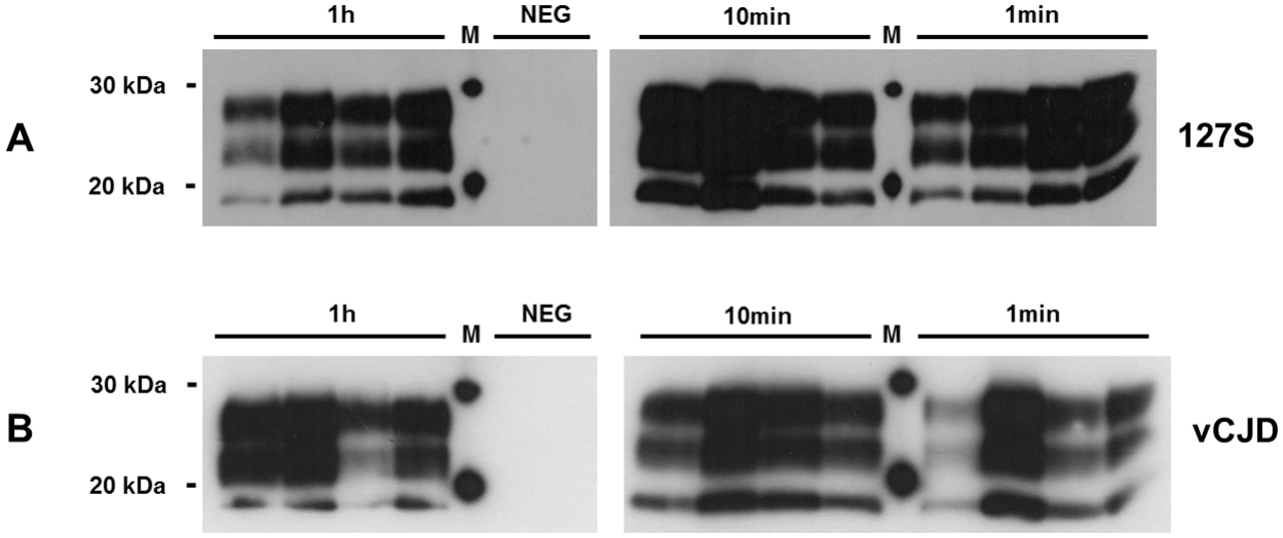
Optimization of steel wire contamination in 96-well microplates. Wires were contaminated with either 1% 127S-scrapie or 10% human vCJD brain homogenates at different times (1 min, 10 min or 1 hr) and subjected to one Surf-PMCA round. Protease-resistant prion protein was detected with 6D11 (127S-scrapie) or 3F4 (human vCJD) monoclonal antibodies. NEG: Negative control (wires mock-contaminated in normal brain homogenate). M: molecular mass marker.

During preliminary experiments, we observed heterogeneous amplification results when wires were grouped together for their contamination with infected brain homogenates (5 wires contaminated in the same tube) prior to Surf-PMCA. Typically, in this batch format only a subset of wires were detected positive for the presence of PrP^TSE^ after one PMCA round, while several others were detected after two or more rounds (data not shown). To overcome this lack of reproducibility, the contamination step was transferred to a microplate format. We incubated the wires in 96-well microplates (one wire per well) in 1% (127S scrapie) or 10% (human vCJD) infected brain homogenates under constant shaking. This new contamination protocol markedly decreased inter-individual variations in Surf-PMCA efficiency. It is noteworthy that it was not possible to detect any PrP^TSE^ on a single wire by Western blot without any amplification (data not shown). All wires contaminated with both 127S scrapie (12/12) and vCJD prions (12/12) were found positive for PrP^TSE^ after the first PMCA round (Fig 1). A contact time of 1 min with both 127S- and vCJD-infected brain homogenates was sufficient to render wires positive following one round of Surf-PMCA. Prolonging the contamination time (10 and 60 min) did not appear to alter the results.

We therefore continued the study using a 96-well microplate format for contamination, rinsing and disinfection studies of wires and set the duration of the wire contamination step to 5 min for practical reasons.

### Determination of Surf-PMCA sensitivity

**Fig 2:**
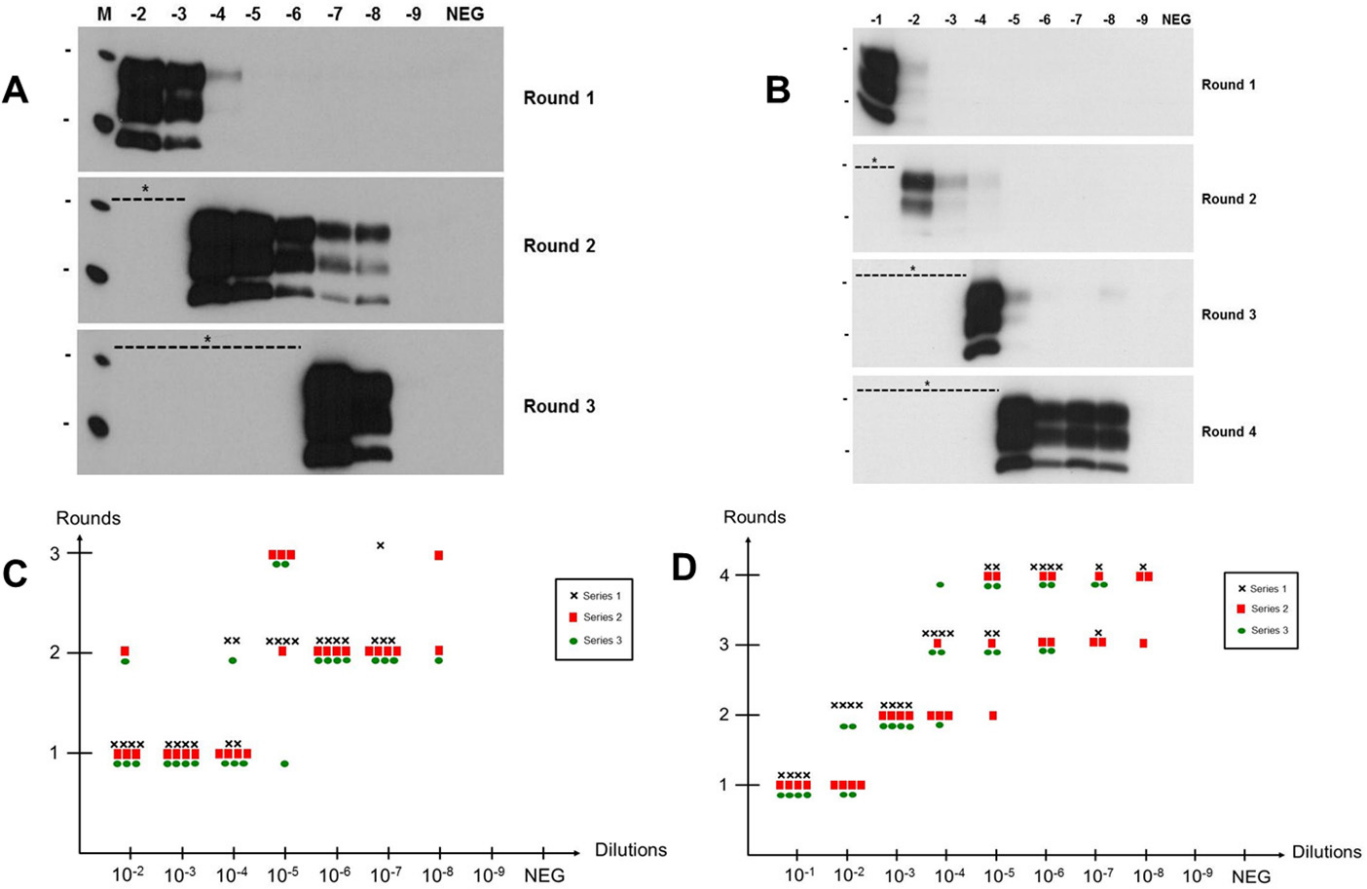
Determination of Surf-PMCA sensitivity. (A and B) Representative Western blot results of serial Surf-PMCA on wires contaminated with serial 10-fold dilutions of 127S (A) or vCJD (B) brain material. (C and D) Individual wires scored positive by serial Surf-PMCA as a function of infectious brain dilution (C, 127S scrapie; D, vCJD) and PMCA round. Three independent Surf-PMCA series were performed with 4 wires per brain dilution (Black cross (**Χ)** Series 1, Red square (■) Series 2 and Green circle (●) Series 3). Protease-resistant prion protein was detected with 6D11 (127S-scrapie) or 3F4 (human vCJD) monoclonal antibodies. NEG: Negative control (wires mock-contaminated in normal brain homogenate). M: molecular mass marker. Bars to the left of Western blot panels indicate the 30 and 20 kilo Dalton marker positions. * Positive wires were not systematically subjected to an additional PMCA round.

To determine the sensitivity of the Surf-PMCA method, wires were contaminated with serial 10-fold dilutions of either 127S or vCJD brain material (from 10^−1^ or 10^−2^ to 10^−9^) and subjected to Surf-PMCA. For each dilution, groups of 12 wires were analyzed in 3 independent series. In order to achieve a high amplification efficiency required for such sensitive monitoring in prion disinfection studies, three to four successive PMCA rounds (depending on the prion strain) were performed into fresh substrate with a 1:10 dilution factor between each round. As shown in Fig 2, out of 24 negative control wires mock-contaminated with normal brain homogenates none achieved a positive PrP^TSE^ signal after three or four rounds of PMCA. This proves that the Surf-PMCA method is 100% specific under the experimental conditions used.

For 127S scrapie prions (Fig 2A and 2C and Table 2), wires contaminated with 10^−2^, 10^−3^ and 10^−4^ dilutions were detected positive for PrP^TSE^ after one PMCA round (31/36). After two rounds, wires exposed to 10^−5^, 10^−6^ and 10^−7^ brain dilutions were detected positive (28/36). Finally, it was possible to detect PrP^TSE^ on 3 out of 12 wires contaminated with a 10^−8^ brain dilution after three PMCA rounds.

**Table 2:** Summary of PrP^TSE^ detection data on wires contaminated with serial dilutions of infectious brain material by Surf-PMCA.

Brain dilution	127S scrapie^a^	Human vCJD^b^
10^−1^	-	12/12
10^−2^	12/12	12/12
10^−3^	12/12	12/12
10^−4^	12/12	12/12
10^−5^	11/12	12/12
10^−6^	12/12	12/12
10^−7^	11/12	7/12
10^−8^	3/12	4/12
10^−9^	0/12	0/12
NEG	0/12	0/12

^a^ Results obtained after three serial PMCA rounds

^b^ Results obtained after four serial PMCA rounds

NEG: Wires mock-contaminated in normal brain homogenate.

For human variant CJD prions (Fig 2B and 2D and Table 2), the results were more heterogeneous. Indeed, after the first Surf-PMCA round, 100% (12/12) of wires contaminated with a 10^−1^ vCJD brain dilution were found positive for PrP^TSE^, whereas only 50% (6/12) of the 10^−2^ contaminated wires gave a positive signal. After the second PMCA round, the remaining 10^−2^ vCJD contaminated wires were positive (6/6) in addition to all 10^−3^ contaminated wires (12/12), as well as 4/12 and 1/12 of wires contaminated with 10^−4^ and 10^−5^ dilutions, respectively. After the third and fourth PMCA rounds positive results were achieved ranging from 10^−4^ to 10^−8^ contaminated wires. Taken as a whole, after four PMCA rounds we found the following proportions of wires positive for PrP^TSE^: 100% (12/12) of 10^−1^ to 10^−6^ dilutions, 58% (7/12) of 10^−7^ and 33% (4/12) of 10^−8^.

Overall, the Surf-PMCA assay detected PrP^TSE^ from both prion strains with a high level of sensitivity, on a single wire and over a large dynamic range (6 to 7 logs).

### Evaluation of "standard" decontamination procedures using 127S scrapie prions

"Standard" decontamination procedures including partially and fully efficient treatments (Table 1) were evaluated on 127S scrapie prions for their efficiency against prions. A control experiment was first designed to check the absence of non-specific background caused by any chemicals that could interfere with the efficacy of the Surf-PMCA assay. Mock-contaminated wires in normal brain homogenate were treated with either sodium hydroxide (1M NaOH—60 min) or sodium hypochlorite (2% NaOCl– 60 min) as harsh chemicals and rinsed, or only water-rinsed (control), and then placed into PMCA reaction tubes containing either a 10^−5^ or 10^−6^ dilution of 127S scrapie-infected mouse brain homogenate (S1 Fig). Similar amplification performances were observed for the chemical treated wires and the control wires, indicating that there was no inhibitory “salting out” effect of residual chemicals on Surf-PMCA efficacy.

We next contaminated wires with 1% 127S scrapie prions and treated them according to various standard decontamination procedures. Two inefficient treatments, water and peracetic acid (PAA), were used as controls. After washing, the residual prion seeding activities on treated wires were investigated by Surf-PMCA. We performed three independent series of Surf-PMCA for each treatment (4 wires per series).

**Fig 3:**
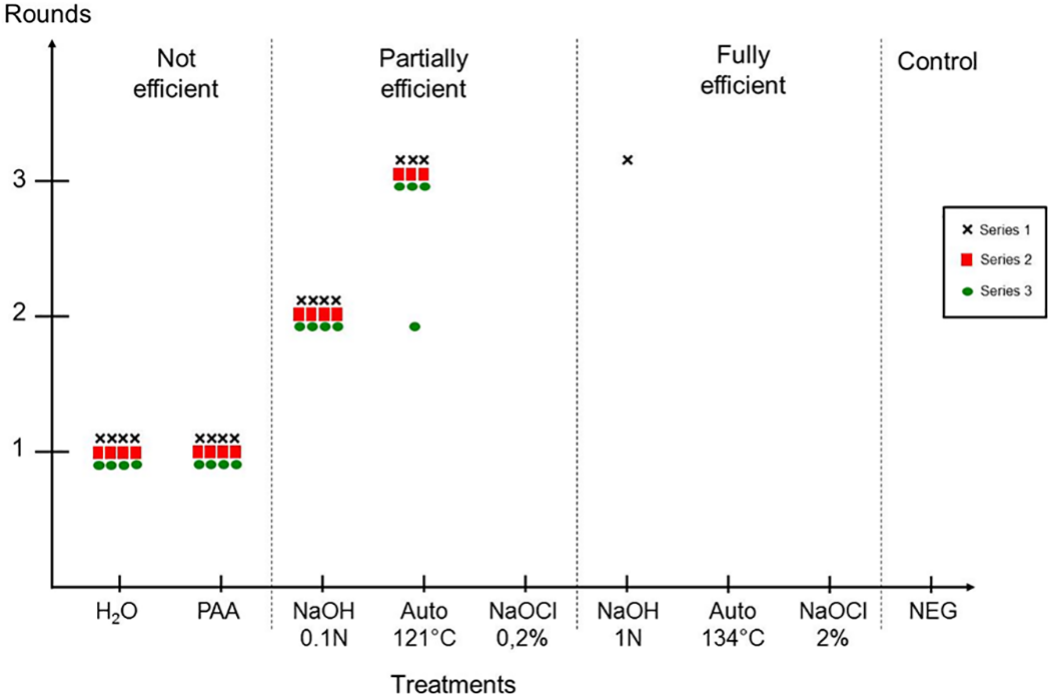
Evaluation of “standard” decontamination procedures on 127S scrapie prions. Steel wires contaminated with 1% 127S scrapie brain homogenate were treated with “standard” prion decontamination procedures and subjected to three serial PMCA rounds. Three independent series of PMCA were performed on 4 wires per treatment (Black cross (**Χ)** Series 1, Red square (■) Series 2 and Green circle (●) Series 3). PAA: Per acetic acid 1.2%—60 min; NaOH: sodium hydroxide 0.1N—15 min or 1N—60 min; Auto: steam sterilization 121°C -20 min or 134°C -20 min; NaOCl: Sodium hypochlorite 0.2% (2000ppm)– 15 min or 2% (20000ppm)– 60 min; H_2_O: untreated positive control; NEG: negative control (wire mock-contaminated with normal brain homogenate).

Fig 3 shows the results obtained after one to three rounds of PMCA. As previously observed, out of the 12 negative control wires mock-contaminated with normal brain homogenates none gave a positive signal for PrP^TSE^, confirming the 100% specificity of Surf-PMCA. As expected, control wires that were either water rinsed or treated with PAA were positive (12/12) after one PMCA round. NaOH at 0.1N (partially efficient treatment) led to an amplification of PrP^TSE^ detectable after two rounds (12/12). By comparing this result with those obtained with the serial dilutions, it suggests that 0.1N NaOH was able to decrease the level of 127S scrapie prion by at least 3 logs. Similarly, after autoclaving at 121°C, 83% of the wires were positive after three rounds (10/12), corresponding to a reduction of at least 5 logs. By contrast, the NaOCl treatment at 0.2% for 15 min which is considered to be partially efficient, led to a complete removal of seeding activity on 127S wires (0/12). Interestingly, only 1 out of 12 wires following the ‘fully efficient treatment’ of 1N NaOH was detected as positive after three PMCA rounds, indicating that the 127S scrapie prion strain is not completely inactivated by 1N NaOH for 60 min. However, we found no positive wires after three PMCA rounds following either steam autoclaving at 134°C or NaOCl at 2% for 60 min, therefore these procedures could be considered as fully efficient.

### Assessment of human vCJD prions inactivation by Surf-PMCA

Due to limited access to human brain homogenates (either vCJD or control) for ethical reasons, only two independent series of Surf-PMCA were performed for the assessment of human vCJD prion inactivation. Nevertheless, concerning the evaluation of marketed reagents, we focused on vCJD prions which are of concern to healthcare facilities as a potential source of iatrogenic transmission.

The decontamination procedures we tested on vCJD prions are summarized in Table 1 and included both "standard" procedures and six anonymized marketed reagents previously validated by the French regulation agency (*A* to *F*) (S1 Table), two of which were tested under two different conditions. After contamination with 10% vCJD prions, wires were treated according to the decontamination procedures and subjected to Surf-PMCA for investigation of residual prion seeding activity. We performed two independent series of Surf-PMCA for each treatment (4 wires per series).

**Fig 4:**
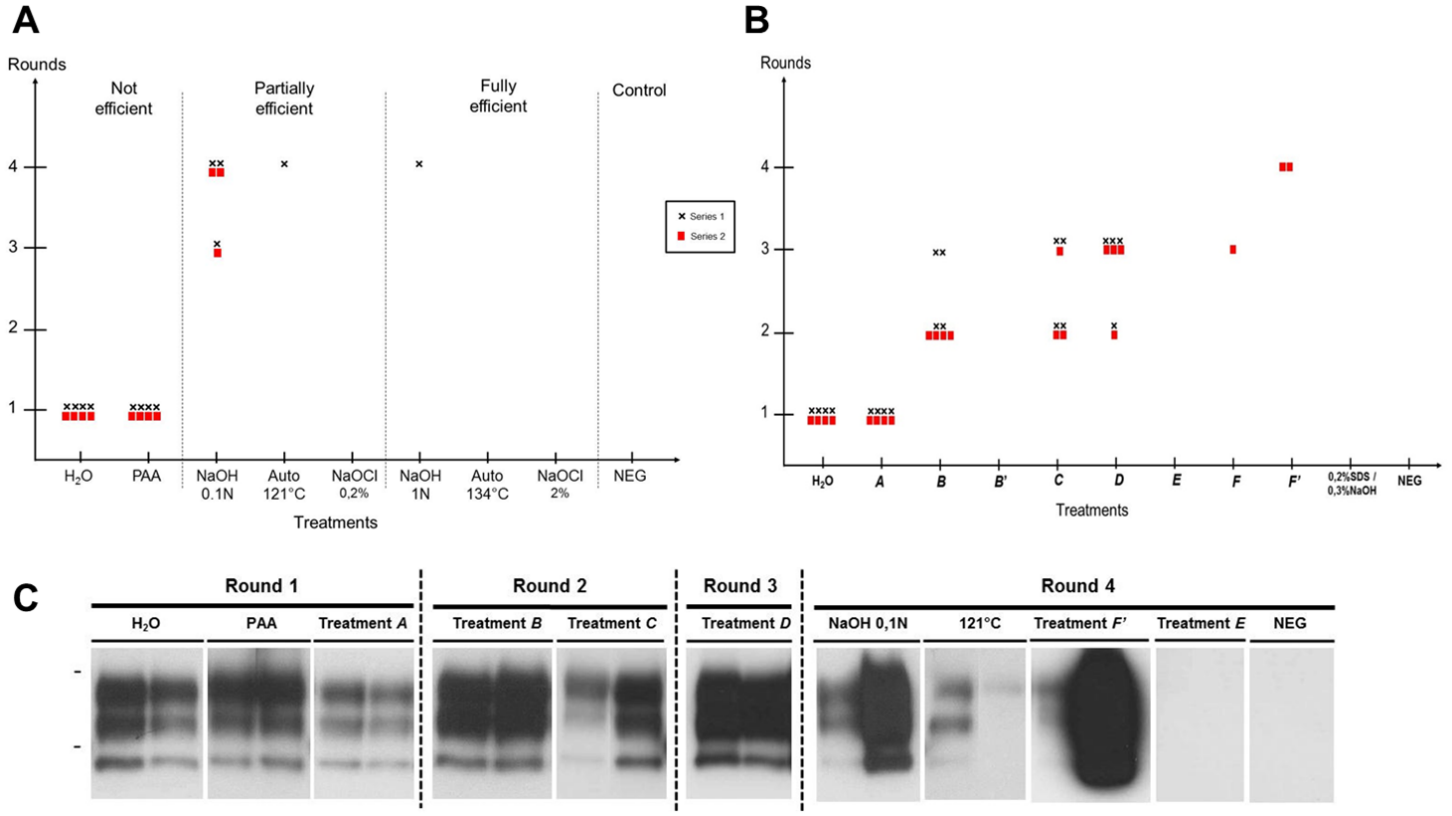
Evaluation of “standard” and commercially available decontamination procedures on human vCJD prions. Steel wires contaminated with 10% human vCJD brain homogenate were treated with “standard” (A) and commercially available (B) prion decontamination procedures and subjected to 4 serial PMCA rounds. Two independent series of PMCA were performed on 4 wires per treatment (Black cross (**Χ)** Series 1 and Red square (■) Series 2). PAA: Per acetic acid 1.2%—60 min; NaOH: sodium hydroxide 0.1N—15 min or 1N—60 min; Auto: steam sterilization 121°C -20 min or 134°C -20 min; NaOCl: Sodium hypochlorite 0.2% (2000ppm)– 15 min or 2% (20000ppm)– 60 min; H_2_O: untreated positive control; NEG: negative control (wire mock-contaminated with normal brain homogenate). (C) Representative Surf-PMCA results from two individual wires after both reference and marketed decontamination procedures (3F4 monoclonal antibody). Bars to the left of Western blot panels indicate the 30 and 20 kilo Dalton marker positions.

Results obtained for the 16 negative control wires mock-contaminated with normal human brain homogenate confirmed the 100% specificity of Surf-PMCA on human samples after 4 rounds (Fig 4A). As previously observed with 127S-scrapie prions, inefficient treatments (water and PAA) led to PrP^TSE^ detection on all vCJD-contaminated wires (8/8) after one PMCA round (Fig 4A). Also, we found that NaOCl treatment, even at 0.2% for 15 min, was fully efficient in decontaminating vCJD wires (0/8). Wires treated with 0.1N NaOH were detected as PrP^TSE^ positive after the third and fourth PMCA rounds (6/8 at final), representing a reduction of around 3 logs. In contrast, only 1/8 wires were positive after four PMCA rounds following steam sterilization at 121°C, corresponding to a reduction of at least 5 logs. Of those procedures considered to be fully efficient, only 1/8 wires treated with NaOH at 1N for 1hr gave a positive signal after four PMCA rounds, and no positive wires were detected after steam autoclaving at 134°C for 20 min.

We then challenged six marketed reagents (Table 1, *A* to *F*) on vCJD-contaminated wires according to recommendations of manufacturers, with two different conditions for two reagents (*B* and *F*). In addition, we tested a 0.2% SDS / 0.3% NaOH treatment, previously described as very efficient on hamster 263K prions [25].

After one PMCA round we detected PrP^TSE^ on 100% (8/8) of vCJD-contaminated wires subjected to Treatment *A*, similar to control wires treated with water or PAA (Fig 4B). Representative Surf-PMCA results from two individual wires following the different treatments are shown in Fig 4C. Treatment *B* at 1X (recommended concentration by the manufacturer) resulted in 8/8 wires with positive signals after three PMCA rounds, whereas at 2X (Treatment *B’*) it efficiently removed vCJD prions from the steel surface (0/8 after four rounds). After three PMCA rounds, treatments *C* and *D* led to 7/8 and 8/8 positive signals, respectively. According to our results, treatment *E* could be considered as fully efficient since 0/8 wires contaminated with vCJD showed detectable PrP^TSE^ after four rounds of PMCA. Treatments *F'* and *F* tested at 1% and 2% (with different treatment parameters—contact time, temperature, stirring), resulted in some positive signals (2/8 and 1/8 respectively) after four PMCA rounds. Finally, treatment with 0.2% SDS / 0.3% NaOH efficiently removed vCJD abnormal prion protein on all wires (0/8).

## Discussion

Prions are exceptionally resistant to conventional hospital sterilization methods [17]; they therefore pose a serious challenge for healthcare facilities to control infection. Similar to conventional infectious agents such as viruses and bacteria, the final validation of inactivation studies aimed at eliminating prion infectivity is dependent on the availability of sensitive diagnostic tools. Inactivation of prions on re-usable medical devices is currently performed using products or procedures that have been validated with bioassay infectivity assays using non-human prion strains, i.e. hamster- or mouse-adapted scrapie prions. However, studies have shown that inactivation procedures validated on rodent prions cannot be fully extrapolated to inactivation of human prions [18, 19]. Among human prions, vCJD prions are considered at risk of iatrogenic transmission due to their widespread tissue distribution [12–14], the existence of asymptomatic infected individuals [30, 31], and the uncertainties surrounding the prevalence of the disease in the general population, particularly in the UK [15, 32].

A major and recurrent limitation in studies aimed at validating decontamination procedures is the lack of a sensitive model succumbing rapidly to human prions. Although bioassays in transgenic mice overexpressing human or bovine PrP [33, 34] have proved to be considerably more sensitive than conventional RIII mouse bioassays for vCJD agent titration [12, 35], their use is nonetheless time-consuming and expensive. This precludes the development and validation of inactivation procedures against this prion strain.

In the present study, our aim was to develop a rapid and cost-effective *in vitro* assay for the detection of human vCJD abnormal prions bound to steel surfaces. Stainless-steel wires are typically used as carrier models of prions for inactivation studies [27–29] by mimicking the surface of surgical instruments. Also, it has been shown that while some procedures significantly reduce prion titers in brain homogenates, their effect on prions bound to the surface of stainless steel wires is limited [18, 19].

Our unique Surf-PMCA assay combines the capacity of PMCA technology to propagate PrP^TSE^ and infectivity *in vitro* [20, 21], with prion-contaminated steel wires as the source of seeding infectious material. We have demonstrated that the adapted Surf-PMCA assay can sensitively detect both ovine 127S scrapie- and human vCJD-associated seeding activity present on a unique wire with 100% specificity and within an approximate timeframe of two weeks. This assay can detect abnormal prion protein in 10^8^-fold dilutions of infected brain homogenate from either strain on a single wire, revealing a greater sensitivity than endpoint infectivity bioassays, which usually have a detection limit of 10^6^- to 10^7^-fold brain dilutions for the same prion strains [33, 36]. Nevertheless, the sensitivities of the Surf-PMCA assay were slightly lower compared to those obtained under standard PMCA conditions for the same prion strains in our laboratory: 10^9^-fold brain dilutions detected for both strains in only two and three rounds for 127S scrapie and vCJD, respectively (data not shown). Prolonging the number of Surf-PMCA rounds could help to assess whether the limiting dilution of scrapie material has been reached under the experimental conditions used in this study. Overall, the Surf-PMCA method has an extremely high level of sensitivity for detection of PrP^TSE^ bound to wires. Most importantly, the ability of the assay to detect genuine human vCJD PrP^TSE^ opens up possibilities for direct testing of decontamination reagents against a range of specific human prion strains, while limiting the use of animals.

We initially determined whether the assay could discriminate between fully and partially effective reference decontamination procedures on wires contaminated with 127S ovine scrapie and human vCJD prions. As expected, those reference procedures considered to be fully effective (steam autoclaving at 134°C, NaOH at 1M and NaOCl at 2%) led to a complete removal of prion seeding activity, except for 1/12 wires contaminated with both prion strains and treated with NaOH at 1M. Following two of the partially effective procedures (steam autoclaving at 121°C and NaOH at 0.1M) we detected positive wires with both prion strains, although at a lesser extent for human vCJD prions. This result was surprising as the BSE/vCJD strain is reputed to be more resistant to heat inactivation than other prion strains [35], and the drying of infected material onto surfaces supposedly increases the thermostability of prions. Further analyses, including the assessment of dry heat inactivation, will be useful to clarify this issue. However, it is noteworthy from the dilution range experiments that one more Surf-PMCA round is required for the vCJD strain to achieve a sensitivity similar to that obtained with 127S prions. This possibly reflects a better propensity of the ovine scrapie strain to be amplified in our assay and/or differences in the amount of misfolded PrP^TSE^ molecules initially present in seed inputs that are able to initiate reactions. Nevertheless, sodium hypochlorite proved to be totally efficient on both prion strains, even at a low concentration (0.2% NaOCl).

We then sought to assess the effectiveness of six marketed reagents to inactivate prions on vCJD-contaminated wires. In addition, we tested a treatment composed of 0.2% SDS and 0.3% NaOH, previously described as being fully efficient on hamster prions [25]. Unexpectedly, among these commercial formulations, only one product was found to be completely efficient in removing vCJD-associated seeding activity. More disturbingly, in our hands, one treatment could be considered as inefficient on vCJD as 100% of wires tested were detected as positive after the first Surf-PMCA round, similar to control-wires treated with water only. Overall, our results indicate that the Surf-PMCA assay is sensitive enough to produce qualitative data to describe the efficiency of procedures for decontamination of the human vCJD prion strain. It will be of great benefit to adapt this detection assay to other types of human prions, in particular sporadic CJD prions.

PMCA has previously been successfully used as a rapid test for the assessment of prion inactivation [37–39]. Recently, the association of PMCA with steel wires was described by Beekes *et al* [25]. The authors initially observed a 7 log reduction in seeding activity of hamster 263K prions on wires via PMCA, while the detection limit of the 263K hamster bioassay using wires is around 5.5 log [29, 40, 41]. In a more recent paper [42] Beekes *et al* adapted their assay allowing the detection of human vCJD abnormal prions on 15 wires (of 5x0.25 mm) in 10^5^-fold brain dilutions after nine rounds of PMCA. When compared to the Surf-PMCA assay which achieved positive detection in a 10^8^-fold brain dilution following only four PMCA rounds on a single wire (of 3x0.15 mm), the gain in sensitivity is clearly substantial. In addition, our results can be directly compared to those of bioassay infectivity studies, as one wire is required for each type of assay.

An important question in line with a risk assessment is whether the prion seeding activity detected by PMCA is a surrogate of the biological infectivity as measured by bioassays. Although occasionally debated [43, 44], a large body of evidence indicates a direct correlation between infectivity and the PMCA seeding activity [36, 37, 39, 45–48]. PMCA was also demonstrated as more sensitive than bioassays by several logs of magnitude for the detection of prions [36, 46, 49]. This extended limit of detection might originate from an absence of prion clearance mechanisms in PMCA reactions [50]. Moreover, PMCA-generated prions appear to be highly infectious, with titers similar, if not identical, to those derived from laboratory bioassays at the terminal stage of the disease [36, 51]. For instance, a complete titration experiment performed on PMCA amplicons demonstrated a strong congruency between the seeding activity and infectivity using the same 127S scrapie strain analyzed in our study [36]. In order to verify whether Surf-PMCA data, derived from using steel wires contaminated with vCJD, correlate with the detection of *in vivo* infectivity over a certain dynamic range (in terms of lethal dose 50 and seeding dose 50), our next study involves intracerebrally implanting end-point dilutions or decontaminated steel wires into susceptible transgenic mice. However, due to the higher sensitivity of PMCA compared to an *in vivo* bioassay, a potential disconnection could occur between *in vitro* and *in vivo* results for those wires which scored positive after prolonged Surf-PMCA rounds (either from dilution range experiments or after certain treatments). Conversely, a negative Surf-PMCA result obtained after a particular treatment would likely indicate a low probability of detecting any residual infectivity in a bioassay, attesting the efficiency of such a decontamination procedure.

Although additional experiments using larger cohorts of wires are required for standardization and statistical validation of a quantitative detection assay, our results show that Surf-PMCA can be used as a rapid, ultrasensitive method for the detection of human vCJD prions adsorbed onto a metallic surface, which will facilitate future development and validation of decontamination procedures against human prion strains without extensive use of animals.

## Supporting Information

S1 Fig No effect of residual chemical compounds on Surf-PMCA. Wires were first mock-contaminated in 10% tg338 normal brain homogenate and treated with NaOCl at 2% or NaOH at 1N or H_2_O only. After rinsing, these wires were placed into PMCA reaction tubes containing a 10^−5^ or 10^−6^ dilution of a 10% 127S-scrapie infected mouse brain homogenate for prion amplification (one PMCA round). Protease-resistant prion protein was detected with 6D11 antibody. -2F: 10^−2^ dilution of 10% 127S-scrapie brain homogenate without PMCA amplification. TP: PMCA positive control. NEG: PMCA substrate only (tg338 normal brain homogenate). Bars to the left indicate the 40, 30 and 20 kilo Dalton marker positions.
(DOCX)

S1 Table List of prion inactivating reagents used in this study and operating conditions. Reagents have been validated by the French regulation Agency (ANSM) (version of 4/4/2012).
(DOCX)
