# Supplementary figures and images for: Rapid and Highly Sensitive Detection of Variant Creutzfeldt - Jakob Disease Abnormal Prion Protein on Steel Surfaces by Protein Misfolding Cyclic Amplification: Application to Prion Decontamination Studies
Source: PLoS One. 2016 Jan 22;11(1):e0146833. doi: 10.1371/journal.pone.0146833 (PMC4723062; doi:10.1371/journal.pone.0146833)

**Supporting Information**

**
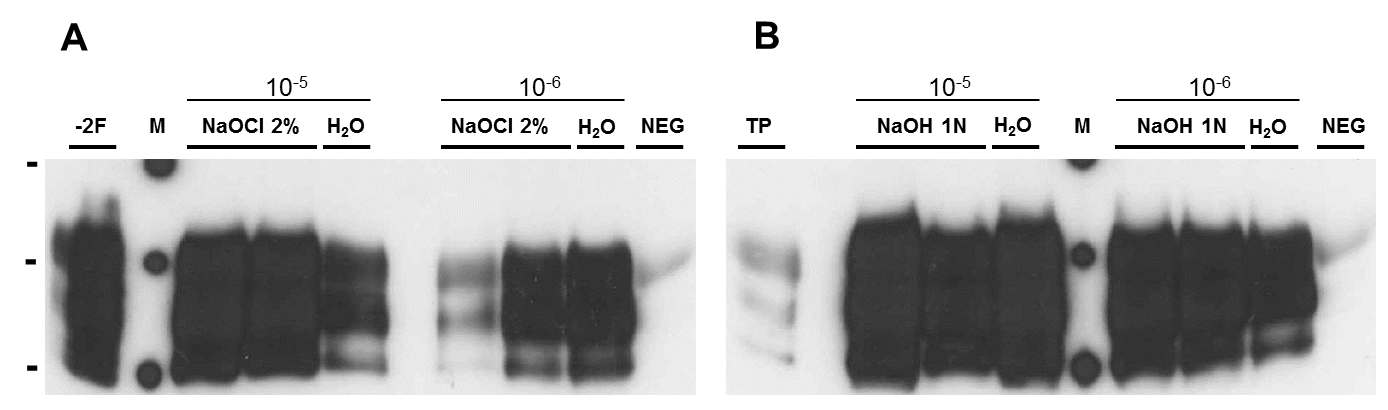
**

**S1 Fig. No effect of residual chemical compounds on Surf-PMCA.**

Supplement: S1 Fig — Wires were first mock-contaminated in 10% tg338 normal brain homogenate and treated with NaOCl at 2% or NaOH at 1N or H2O only. After rinsing, these wires were placed into PMCA reaction tubes containing a 10−5 or 10−6 dilution of a 10% 127S-scrapie infected mouse brain homogenate for prion amplification (one PMCA round). Protease-resistant prion protein was detected with 6D11 antibody. -2F: 10−2 dilution of 10% 127S-scrapie brain homogenate without PMCA amplification. TP: PMCA positive control. NEG: PMCA substrate only (tg338 normal brain homogenate). Bars to the left indicate the 40, 30 and 20 kilo Dalton marker positions. (DOCX) [file pone.0146833.s001.docx]
