# Supplementary material for: Rapid and Highly Sensitive Detection of Variant Creutzfeldt - Jakob Disease Abnormal Prion Protein on Steel Surfaces by Protein Misfolding Cyclic Amplification: Application to Prion Decontamination Studies
Source: PLoS One. 2016 Jan 22;11(1):e0146833. doi: 10.1371/journal.pone.0146833 (PMC4723062; doi:10.1371/journal.pone.0146833)
Supplement: S1 Table — Reagents have been validated by the French regulation Agency (ANSM) (version of 4/4/2012). (DOCX) [file pone.0146833.s002.docx]

**Supporting Information**

**S1 Table. List of prion inactivating reagents used in this study and operating conditions.**

| **Product Name** | **Manufacturer** | **Operating conditions** |
| --- | --- | --- |
| **ACTANIOS HLD** | ANIOS | Immersion / ready to use / RT / 30 min |
| **ACTANIOS HLD**  **+ ACTANIOS P1**  **+ACTANIOS P2** | ANIOS | -P1: immersion/ 0.5% / RT / 10 min  -Rinsing  -P2: immersion/ 0.5% / RT / 5 min  - Rinsing  -HLD: immersion / ready to use / RT / 15 min |
| **ACTANIOS LDI** | ANIOS | [Washer-disinfectors](http://www.getinge.com/healthcare/products/cleaning-disinfection/washer-disinfectors/), sterilizing tunnel / 1% / 55°C / 10 min |
| **ALKA 100** | ALKAPHARM | Immersion / 1% RT / 15 min |
| **HAMO 100** | STERIS | Immersion, [Washer-disinfectors](http://www.getinge.com/healthcare/products/cleaning-disinfection/washer-disinfectors/), sterilizing tunnel / 0.8% / 43°C / 7.5 min |
| **NEODISHER**  **SEPTOCLEAN** | DR WEIGERT | -Immersion / 1% / RT / 60 min  -[Washer-disinfectors](http://www.getinge.com/healthcare/products/cleaning-disinfection/washer-disinfectors/), sterilizing tunnel / 1% / 55°C / 10 min |

Reagents have been validated by the French regulation Agency (ANSM) (version of 4/4/2012)

RT: Room temperature
